# AGE-FRIENDLY CAMPUS PRACTICES IN HIGHER EDUCATION: A HEAT MAP VIEW

**DOI:** 10.1093/geroni/igac059.1719

**Published:** 2022-12-20

**Authors:** Janelle Fassi, Celeste Beaulieu, Lauren Bowen, Joann Montepare, Susan Whitbourne, Nina Silverstein

**Affiliations:** University of Massachusetts Boston, Goffstown, New Hampshire, United States; University of Massachusetts Boston, Boston, Massachusetts, United States; University of Massachusetts Boston, Boston, Massachusetts, United States; Lasell University, Newton, Massachusetts, United States; University of Massachusetts Amherst, Amherst, Massachusetts, United States; University of Massachusetts Boston, Boston, Massachusetts, United States

## Abstract

The Age-Friendly University (AFU) initiative aims to increase the participation of age-diverse older adults in higher education communities. The present study investigated age-friendly practices across 23 institutions in the United States. The ICCS Inventory (Silverstein et al., 2022), which identifies 192 potential age-friendly campus practices was completed by administrators representing major campus units. A heat map was used to graphically represent age-friendly practices and identify where universities differed in the presence of those practices. Heat map findings indicated campuses are low in some auxiliary services that assist retired faculty and staff. However, campuses consistently gave retired faculty and staff access to university library services. Campuses also had limited age-friendly teaching and learning services. None of the campuses reported having resources to help faculty deliver teaching materials in formats specifically geared toward older learners. In addition, none of the campuses reported having teaching and learning staff visiting campus departments to provide resources for older learners, and very few campuses offered courses that focused on aging and age diversity issues. Common age-friendly practices were seen with respect to providing instructional technology support for faculty/staff/students and community partnerships for intergenerational activities. Physical environment and personnel evidenced the most frequent age-friendly practices likely because they are mandated by the ADA (e.g., clear signage, handicapped parking close to buildings, spaces free of obstacles, training in ageism as a form of discrimination). Overall, the present study highlighted the areas where college campuses are most age inclusive, while also revealing areas for improvement in age inclusive practices.

